# Investigation of bioactive compounds from *Bacillus* sp. against protein homologs CDC42 of *Colletotrichum gloeosporioides* causing anthracnose disease in cassava by using molecular docking and dynamics studies

**DOI:** 10.3389/fmolb.2022.1010603

**Published:** 2022-09-23

**Authors:** Narendra Kumar Papathoti, Kishore Mendam, Bala Hanumath Sriram Kanduri, Wannaporn Thepbandit, Rungthip Sangpueak, Chanon Saengchan, Nguyen Huy Hoang, Vineela Sai Megavath, Madhuri Kurakula, Toan Le Thanh, Natthiya Buensanteai

**Affiliations:** ^1^ School of Crop Production Technology, Suranaree University of Technology, Nakhon Ratchasima, Thailand; ^2^ Department of Zoology, Dr. B.R. Ambedkar Open University, Hyderabad, Telangana, India; ^3^ R&D Division, Sri Yuva Biotech Pvt Ltd., Hyderabad, Telangana, India; ^4^ Department of Biotechnology, Mahatma Gandhi University, Nalgonda, Telangana, India; ^5^ Department of Plant Protection, Can Tho University, Can Tho City, Viet Nam

**Keywords:** *Colletotrichum gloeosporioides*, Cdc42, Manihot esculenta, docking, molecular dynamics simulation

## Abstract

*Manihot esculenta*, commonly called cassava, is an economically valuable crop and important staple food, grown in tropical and subtropical regions of the world. Demand for cassava in the food and fuel industry is growing worldwide. However, anthracnose disease caused by *Colletotrichum gloeosporioides* severely affects cassava yield and production. The bioactive molecules from *Bacillus* are widely used to control fungal diseases in several plants. Therefore, in this study, bioactive compounds (erucamide, behenic acid, palmitic acid, phenylacetic acid, and β-sitosterol) from *Bacillus megaterium* were assessed against CDC42, a key protein for virulence, from *C. gloeosporioides*. Structure of the CDC42 protein was generated through the comparative homology modeling method. The binding site of the ligands and the stability of the complex were analyzed through docking and molecular dynamics simulation studies, respectively. Furthermore, a protein interaction network was envisaged through the STRING database, followed by enrichment analysis in the WebGestalt tool. From the enrichment analysis, it is apparent that bioactive from *B. megaterium* chiefly targets the MAP kinase pathway that is essential for filamentous growth and virulence. Further exploration through experimental studies could be advantageous for cassava improvement as well as to combat against *C. gloeosporioides* pathogen.

## Introduction

Plants are an important source for humans, animals, birds, and other living organisms. Plants protect themselves from a variety of biotic and abiotic stresses (Gong et al., 2020). Biotic stress occurs due to bacterial and fungal pathogens (Peck and Mittler, 2020). One of the important plants on which more than 800 million people worldwide are depending for major food sources is *Manihot esculenta* Crantz (Atwijukire et al., 2019). The crop, commonly known as cassava, is enriched with several nutrients such as starch, carotenoids, vitamins, and minerals. Cassava is consumed as the primary food source mainly in the regions of tropical and sub-tropical countries. Later, due to increased industrial importance such as the production of animal feed, biomedicine, and cosmetics, the production of cassava has been highly increased (Li et al., 2017). In addition, the raw materials from *M. esculenta* were used for biopolymer, starch, and bioethanol production (El-Sharkawy 2004). Apart, the by-products from the cassava industries are rich with organic residues essential for the production of value-based products (Ayling et al., 2012). Hence, the crop with human value and with the immense industrial application was cultivated by both low-scale and high-scale cultivators. However, the plant is restricted at an economical level due to several factors such as the presence of cyanogen compounds (Balyejusa Kizito et al., 2007), a low level of proteins, and infectious disease.

Anthracnose disease (AD) damages the healthy planting materials of cassava, leading to low yield and total economic loss for the planters. AD occurs in cassava due to the fungal pathogen *Colletotrichum gloeosporioides* f. sp. *manihotis* (Machado et al., 2020). The fungal strain infects the shoot tips of the healthy plants; develops cancerous growth on the stem and leaves. AD is notorious to cause shoot tip-die-back disease because the pathogen infects the stem region, weakens the parts and leads to major destruction during strong wind and rain (Pinweha et al., 2015). The primary interaction of the pathogen with the cassava plant was established by producing an infection cell known as appressorium. The melanized cell surrounding the aspersorium supports the internal solute concentration and rigidity of the cells (Wang et al., 2021). After the interaction with the host, the pathogen develops infection vesicles and primary hyphae. Later, the fungi develop secondary hyphae structures that spread the infection and kill the plants. Generally, after the successful infection into the host, the fungi adapt to the biotrophic mode of nutrition for their survival (Li et al., 2021). The pathogen produces lesions on leaves, stems, and other parts of the plant. Sequentially, switches to the necrotrophic mode of nutrition in which the pathogen absorbs nutrition from the dead cells of the infected region. This nutrition adaptation by the pathogen is known as the hemibiotrophic mode of infection (Jacobs et al., 2019). Thus, it is very challenging to impair the growth and spread of infection by *C. gloeosporioides*. This pathogen also infects humans but knowledge about the type of disease and mode of infection is not clear so far.

To prevent fungal infection, chemical fungicides were widely used to control the disease (Ons et al., 2020). The use of several fungicides has resulted in impacts on human health and environmental issues. Hence, as an alternative approach to overcome AD-mediated damage in the cassava, different novel approaches were promoted for the development of fungal resistant-cassava crops (Koehorst-van Putten et al., 2012). The genetic engineering approach was one of the conventional strategies known to be the most economical, safe, and effective method to generate anthracnose disease-resistance cassava plants (Hormhuan et al., 2020). The use of a conventional breeding strategy with cassava crops leads to high heterozygosity, low fertility, delayed flowering, and prolonged vegetative stage. Hence, the approach of *Agrobacterium*-mediated transformation is considered to improve the acquired resistance in plants. One of the important plant-pathogen resistance genes, transferred into cassava has been reported to show improved resistance against a wide variety of plant pathogens. Thus, alternative strategies were required to incredulous the current scenario of anthracnose disease in cassava plants. The cell division cycle (CDC42) protein present in the fungi performs the function of the molecular switch by regulating signal transduction pathways and cytoskeleton-mediated cellular process. The protein belongs to the Rho-family of the GTP-binding protein, which plays a pivotal role in the transduction of polarity signals for morphogenetic development (Wang et al., 2018). The CDC42 protein also plays an important role in cell differentiation and appressoria development. CDC42 protein reported with plants is highly diverse, however, the protein is conserved in other eukaryotic species. The protein CDC42 from different fungal species (*Magnaporthe grisea*, *Claviceps purpurea,* and *Ustilago maydis*) has a key role in plant-pathogen interaction (Oeser et al., 2017; Zheng et al., 2009). Thus, the deletion of CDC42 from pathogens has significantly reduced the virulence mechanism during infection. Therefore, in the present study, CDC42 of *C. gloeosporioides* was selected as the therapeutic target to screen for inhibitors against the protein. Also, the detailed investigation using a protein-protein interaction network will pave the way to study the characteristic properties of CDC42 involved in the different biological processes of host-pathogen interaction.

## Materials and methods

### Generation of homology model and structure validation

The three-dimensional structure of Cell division control protein 42 homologs (CDC42) from *C. gloeosporioides* was determined through the comparative homology modeling method. The structure of CDC42 was built through the SWISS-MODEL server (https://swissmodel.expasy.org/). The accuracy of the model was assessed by QMEAN4 score analysis (Benkert et al., 2011). Later, energy minimization was performed using the steepest descent algorithm using GROMACS (Van Der Spoel et al., 2005). The structure of the predicted model was assessed through the structure validation tool SAVES v6.0 program - VERIFY 3D (Agarwal et al., 2021; Hasan et al., 2021), ERRAT (Adewole and Ishola, 2021), WHAT CHECK (Sekhar Pagadala et al., 2009), and PROCHECK analysis (Laskowski et al., 1996). The geometry and stereochemistry of the modeled structure were analyzed through the Ramachandran plot analysis method (Agarwal et al., 2021). In addition, structural validation of the generated model was performed through ProSA score analysis (Wiederstein and Sippl., 2007). Then, the overall quality score of the homology model was compared with the score of the template structure.

#### Binding site prediction

Prediction of druggable cavities is a crucial step for structure-based drug designing. The active site as predicted for CDC42 model protein using sitemap. The prediction of the active site reveals the shape, size and chemical interaction of the ligands with the receptor protein.

### Molecular docking

Five monomeric bioactive compounds identified from the ethyl acetate extract of *Bacillus megaterium* erucamide, behenic acid, palmitic acid, phenylacetic acid, and β-sitosterol, were examined against CDC42 protein of *C. gloeosporioides*. The structure of the bioactive compounds was obtained from the PubChem database. The selected ligands were prepared and the three-dimensional (3D) coordinates were generated. For molecular docking, the proteins used for the study were prepared using protein preparation wizard. The proteins were subjected for H-bond optimization. The entire protein structure were relaxed using Uff force field. Energy minimization for protein and ligand was performed before docking using default parameters. Autodock tools were utilized for the addition of hydrogen, Kollman charges, and solvation parameters (Azam and Abbasi, 2013). Molecular docking was performed through the Autodock Vina program (Trott and Olson, 2010). The grid size of 3 Å for the coordinates X, Y, and Z centered at X: 12.20; Y: 5.95; Z: 7.22 with a grid spacing of 0.375 Å was used for the docking program. The pose with the lowest binding energy was selected as the best conformation. The modeled structures were visualized through the BIOVIA Discovery Studio visualizer (Studio, 2015). Molecular mechanics of combined generalized born surface area and surface area continuum solvation (MM/PBSA and MM/GBSA) methods were performed for studying the effectiveness of interaction. The calculation for average binding free energy ΔGbind was represented for estimating the free energy of ligands binding to the macromolecules. During molecular dynamics simulation of the receptor-ligand complex, the molecular mechanics is applied with empirical scoring and perturbation methods for predicting the accuracy during their simulation run. The formula for average binding free energy ΔGbind was calculated as; ΔGbind = ΔEMM+ ΔGSolv+ ΔGSA.

ΔEMM: denotes minimized energies of protein and ligand.

ΔGSolv: solvation-free energy.

ΔGSA: surface area energy.

#### ADMET properties of the ligands

SMILE structure of the lead molecules used for the present study were downloaded from Pubchem database. The pharmacokinetic properties of molecules were predicted using ADMETSAR2.0. The properties such as acute oral toxicity, BBB, fish aquatic toxicity and carcinogenicity of the molecules were analysis.

### Molecular dynamics simulation

For each protein-ligand system, their pose with the lowest binding energy was assessed. The system was minimized and equilibrated under the number of particles, volume, and temperature (NVT) and the number of particles, pressure, and temperature (NPT) conditions. The molecular dynamics simulation was performed for 50 ns in DESMOND with GPU support. The Optimized Potential for Liquid Simulations (OPLS) force field was used. The system was solvated in a dodecahedron box using a simple point charge (SPC) model with a periodic boundary condition. The system was neutralized by adding sodium chloride ions. Energy minimization was performed through the steepest descent algorithm. Harmonic position restraints were applied during the NVT ensemble simulation. The molecular dynamics production runs were carried out at a 2 fs time step. Temperature and pressure were controlled by setting the Langevin dynamics and Berendsen barostat at 300 K and 1 bar, respectively. Standard periodic boundary conditions and cut-off distance (1 nm) were updated. The particle-mesh Ewald (PME) method was used to assess the interactions. The bonds were constrained with a linear constraint solver (LINCS) and the water molecules were constrained with SETTLE (Hess 2008; Tripathi et al., 2022). Molecular dynamics simulation was evaluated using root mean square deviation (RMSD) and hydrogen bond analysis.

### STRING analysis

The interacting proteins of CDC42 were predicted through the STRING database (http://string-db.org) and the network is built by providing the CDC42 protein sequence in the input box. The search was performed against *C. gloeosporioides*. The confidence score was set to high (0.7). The interactions were based on the experiments, co-expression, databases, gene fusion, neighborhood, and co-occurrence. The maximum number of interactions was set to no more than ten in the first and second shells.

### Identification of clusters from the protein-protein interaction network

Clustering of interactions from large protein-protein interaction networks is essential to define the molecular complexes and topological modules. It is difficult to comprehend and interpret the network properties as such in large protein-protein interaction networks. Therefore, clustering of networks is significant in unraveling the pure network properties as well as finding the network connections in the dense regions. The network obtained from the STRING database is a network based interaction evidence for data support. The obtained network was reconstructed in Cytoscape 3.8.0. The constructed network was evaluated further using Molecular Complex Detection (MCODE) plug-in to visualize the central network. The cut off parmaters were set as MCODE score >3 and node number >4. The subclusters generated were further visualzed and group to study the clossness and degree of interaction in their group.

### Gene ontology (GO) analysis and protein interaction analysis

The gene ontology (GO) analysis was performed in which the functional annotation was achieved through DAVID (database for annotation, visualization, and integrated discovery) database. GOView, a web-based WebGestalt (WEB-based GEne SeT AnaLysis Toolkit) application, is used to visualize and compare the interactional relationship in the network (Zhang et al., 2005; Zhang et al., 2004). Furthermore, the central gene sets were annotated and the hierarchical associations were defined. The protein SHO1 involved in the MAPK signaling pathway was modelled using modeler. SHO1 from yeast was used as the template (2vkn. Pdb) with 61.02% with target sequence. The model of SHO1 and CDC42 was loaded into Patchdock server and protein-protein docking was performed (Yousafi et al., 2021). The protein complex was analysed and results were represented.

## Results

### Generation of homology model and structure validation

**FIGURE 1:**
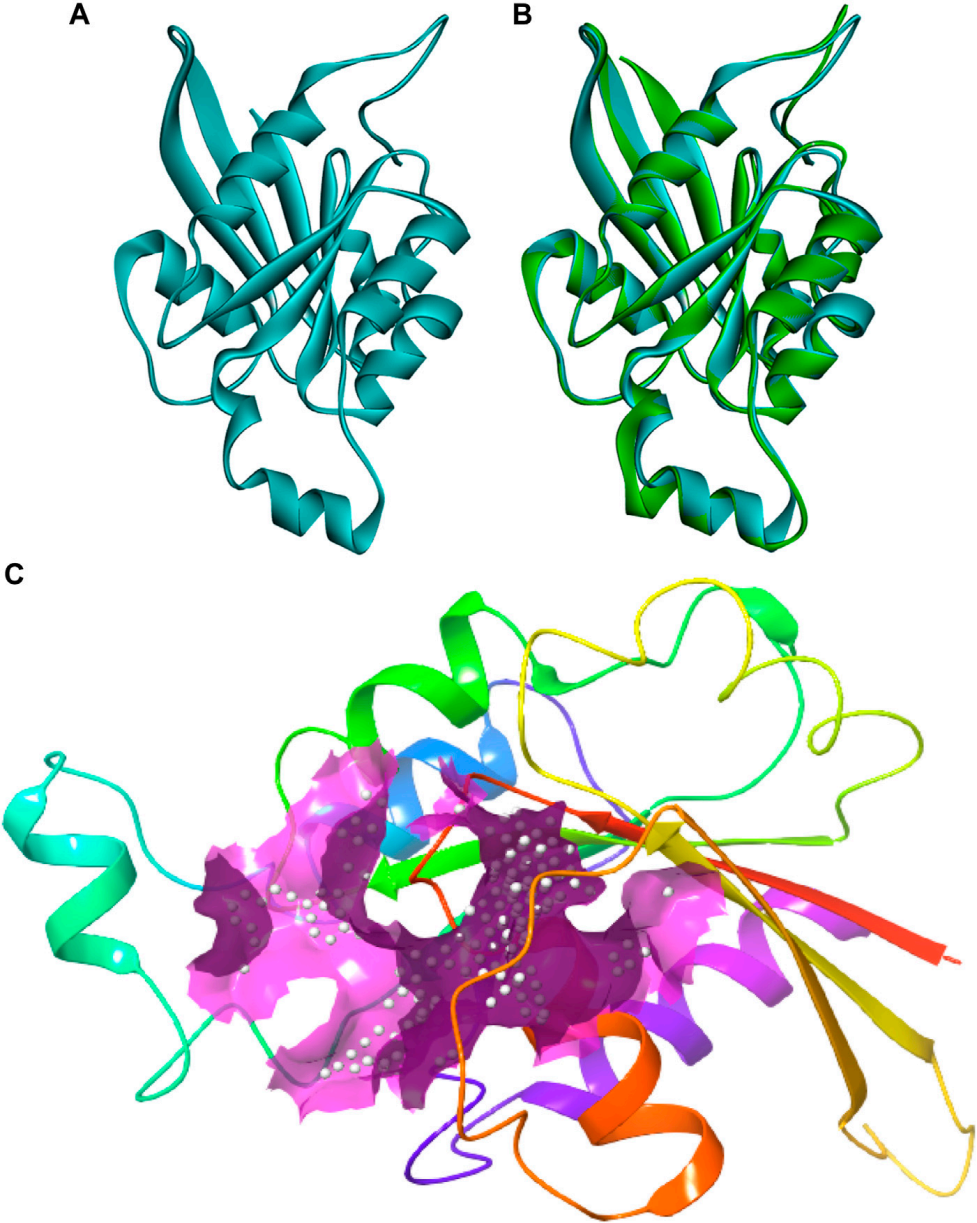
Three-dimensional structure of CDC42 predicted through homology modeling **(B)** The structure of CDC42 protein superimposed with the template (2NGR) structure. **(C)** Binding site prediction representing the active site region (pink).

The protein sequence of the CDC42 homolog of *C. gloeosporioides* (O94103) was retrieved from the NCBI database (https://www.ncbi.nlm.nih.gov/protein/O94103). The sequence contains 190 amino acids and belongs to the small GTPase superfamily, the Rho family. The sequence was predicted to contain three nucleotides (GTP) binding regions: 12–19 (GDGAVGKT), 59–63 (DTAGQ), and 117–120 (TERG). Based on sequence homology, the molecular function involves GTPase activity and the biological process involves cell cycle and cell division. The sequence contains a propeptide region from amino acid 188 to 190 (LVL), which is predicted to be cleaved during protein maturation or activation. The detail for the propeptide region was revealed through the prediction evidence sequence similarity search tool ECO: 0000250 mentioned in the NCBI protein sequence database. The structure of this CDC42 protein was predicted through comparative homology modeling. It showed 70.83% sequence similarity with 98% query coverage with human cell division control protein 42 homolog. The modeled structure was superimposed with the template structure and it is shown in Figure 1. The overall quality factor obtained during ERRAT analysis is 77.5281. In Verify3D, around 80.65% of the amino acid residues have scored≥0.2 in the 3D/1D profile. Ramachandran’s Z-score was found to be −2.077 in the WHAT CHECK analysis. In Ramachandran plot analysis 88.7% amino acid residues were found in the most allowed regions. Around 10.7% residues were present in additionally allowed regions and 0.6% amino acid residues were found in generously allowed regions. The overall quality analyzed through ProSA Z-score displayed a −6.62 value for target CDC42 homology while the template displayed a Z-score value of -7.59. This Z-score comparison between the target and the template suggests the resemblance in the geometry of the conformations between the target and template. Structural validation was shown in (Supplementary Figure S1). Altogether the structural verification suggested the consistency of the generated model.

### Molecular docking

**FIGURE 2:**
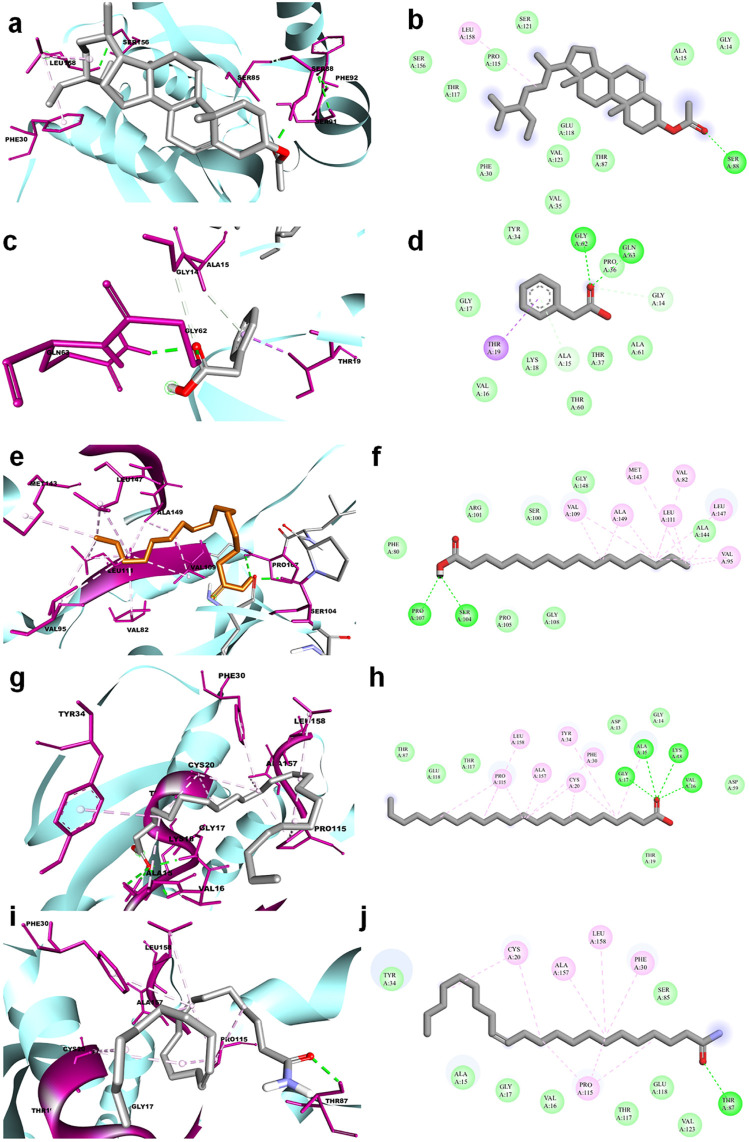
3D and 2D representation of molecular docking of CDC42 with ligands from *B. megaterium*. The ligands and their interaction are shown with the line diagram. The color code green color represents the hydrogen bond. Purple color represents pi-sigma interaction. Light pink color represents, pi-alkyl and alkyl interaction. **(A,B)** = beta sitosterol, **(C,D)** = Phenylacetic acid, **(E,F)** = palmitic acid, **(G,H)** = Behenic acid and **(I,J)** = Eurcamide.

The molecular docking results for active compounds identified from *B. megaterium* were shown in Table 1. Inconsistent with previous reports from Xie et al. (2021), the results from the present study also showed β-sitosterol and phenylacetic acid as the top hits in molecular docking. Through binding site prediction, it was observed that Leu158, Ser121, Thr117, Glu118, Ser88, Thr87, Ala15, Glu18, and Gly14 are the active residues of CDC42. Active site region is distributed with polar (Ser and Thr), hydrophobic residues (Leu and Ala) and negatively charged (Glu) residues. The presence of Ser residues in the active region are responsible for the interaction with the lead molecules. Presence of single Ser residues are responsible for enzymatic reaction. The binding site region consist of two Ser residues responsible for interaction of the lead molecules. Both the ligand, β-sitosterol and phenylacetic acid, presented the highest dock score of −10 kJ mol ^−1^. The next top hit obtained was palmitic acid (−9.4 kJ mol ^−1^) followed by behenic acid (−9.2 kJ mol ^−1^) and erucamide (−9.2 kJ mol ^−1^). The representative 2D and 3D images were presented in Figure 2. The CDC42 homolog protein with phenylacetic acid displayed van der Waals interactions with Gly17, Val16, Lys18, Thr37, Thr60, Ala61, Pro36, Val35, Tyr34; conventional hydrogen bond interactions with Gly62, Gln63; carbon-hydrogen bond and pi-donor hydrogen bond interactions with Ala15, Gly14; and pi-sigma bond interaction with Thr19. The CDC42 protein with β-sitosterol displayed van der Waals interactions with Pro115, Ser121, Ser156, Thr117, Phe30, Val123, Glu118, Thr87, Ala15, and Gly14; conventional hydrogen bond interaction with Ser88; and alkyl bond interaction with Leu158. Palmitic acid displayed van der Waals interactions with Phe80, Pro105, Gly108, Arg101, Ser100, Gly148, Ala144; conventional hydrogen bond interactions with Ser104, Pro107; and alkyl bond interactions with Val109, Ala149, Met143, Leu111, Val82, Leu147, Val95. The CDC42 homolog with behenic acid displayed van der Waals interactions with Thr87, Glu118, Thr117, Asp13, Gly14, Asp59, Thr19; conventional hydrogen bond interactions with Ala15, Val16, Gly17, Lys18; alkyl and pi-alkyl bond interactions with Pro115, Ala157, Leu158, Tyr34, Phe30, Cys20. Erucamide showed van der Waals interactions with Tyr34, Ala15, Gly17, Val16, Thr117, Glu118, Val123, Ser85; (Table 2) conventional hydrogen bond interaction with Thr87; alkyl and pi-alkyl bond interactions with Cys20, Phe30, Pro115, Ala157, Leu158. From the free energy calculation, it was observed that β-sitosterol obtained the highest binding energy of -42.35 (Kcal/mol) compared to the other molecules used for docking. Palmitic acid with an energy of -41.51(Kcal/mol) was observed as second highest compound showing highest binding affinity. The other compounds such as phenylacetic acid, Eurcamide, and Behenic acid was observed with -39.26, -39.49 and -37.90 was observed with binding energy respectively.

**TABLE 1:** Docking score of different ligands from *B. megaterium* with CDC42 protein.

S. No	PubChem ID	Compound name	Dock score (kJ mol ^−1^)	ΔGbind (Kcal/mol)
1	222284	beta-Sitosterol	−10	-42.35
2	999	Phenylacetic acid	−10	-39.26
3	985	palmitic acid	−9.4	-41.51
4	8,215	Behenic acid	−9.2	-37.90
5	5365371	Eurcamide	−9.2	-39.49

**TABLE 2:** GO Slim summary is based on Entrez gene IDs.

S. No	Gene symbol	Gene name	Entrez gene
1	BMH1	14-3-3 family protein BMH1	856924
2	BUD6	Bud6p	851029
3	CLA4	serine/threonine protein kinase CLA4	855418
4	SHO1	osmosensor SHO1	856854
5	SPA2	Spa2p	850639
6	STE50	Ste50p	850325

### ADMET

All the compounds were predicted positive for intestinal absorption and blood brain barrier. Also, from the predicted results it was observed that the compounds were non AMES toxic and non-carcinogenic. Hence the predicted compounds were determined non-toxic and can be used extensively for further studies. Also, β-sitosterol was previously predicted as FDA approved drug with no side-effects (Babu and Jayaraman, 2020). Based on the pharmacokinetic properties, the molecules were predicted to be lead molecules (Table 3).

**TABLE 3:** Predicted ADME physio-chemical properties of the docked compounds, all the tables cited correctly.

Compound name	Human intestinal absorption	BBB	Acute oral toxicity (log (1/(mol/kg))	Fish aquatic toxicity	Carcinogenicity (binary)
Beta sitosterol	0.9930	0.9247	1.989	0.9917	0.9714
Phenylacetic acid	0.9490	0.9659	1.697	0.4220	0.7286
palmitic acid	0.8417	0.9725	1.16	0.9178	0.6571
Behenic acid	0.8417	0.9725	0.6378	0.9178	0.6571
Eurcamide	0.9186	0.9969	0.6537	0.7699	0.6429

### Molecular dynamics simulation

**FIGURE 3:**
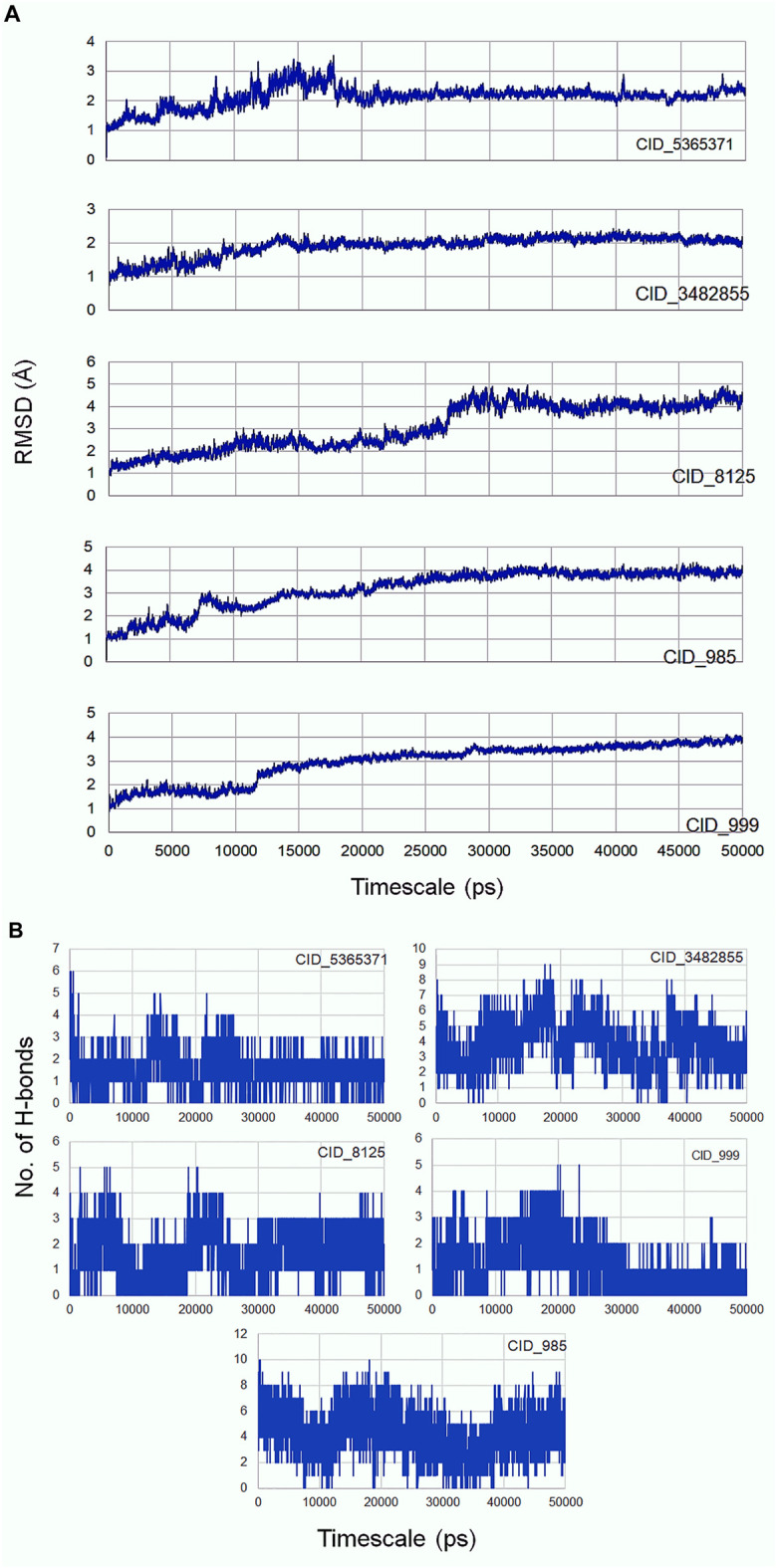
(continued).

Molecular dynamics simulation is an efficacious method for validating the stability of the ligands (β-sitosterol, phenylacetic acid, palmitic acid, behenic acid, and erucamide) docked into the binding pocket of CDC42. For this, all-atom molecular dynamics (MD) simulation study was applied which is regarded as a valuable approach to study the dynamic behavior of the ligands and proteins along with their key interacting residues. Thus, the obtained protein-ligand complexes through molecular docking were enrolled for 50 ns of all-atom MD simulation. MD simulation results revealed the protein-ligands exhibited successful conversion of the initial start of run from 15 ns (Figure 3). The trajectories analysis of the MD run has shown the rise of initial frames at an average of 15 ns. However, the RMSD level of the trajectories proceeded with the average values with minimal fluctuation until 20 ns. The ligand β-sitosterol showing interaction with CDC42 during MD simulation has exhibited an initial rise of the frames from 10 to 15 ns. The standard plateau throughout the MD simulation interval was observed from 20 to 50 ns. The average RMSD for β-sitosterol was observed as 2.10 ± 0.20 Å. This dynamic behavior confers a more stabilized accommodation of β-sitosterol into the binding pocket of the CDC42 throughout the MD simulation. The average RMSD values for phenylacetic acid, throughout the plateau MD simulation interval (12–28 ns) was 3.1 ± 0.50 Å. However, the plateau showed a rise in level after 30 ns and remained stable until 50 ns with an average RMSD of 3.5 ± 0.30 Å. Palmitic acid showed a higher shift in trajectory frames with an RMSD value rise from 3.1 ± 0.50 Å to 4.3 ± 0.10 Å after 25 ns. However, both the ligands phenylacetic acid and palmitic acid have converged around the comparable trajectory frames with an average RMSD value of approximately above 3.5 Å. This dynamic difference between palmitic and phenylacetic acid has shown that the ligands might have deviated from the original interaction compared to the β-sitosterol ligand complex. The other ligands such as behenic acid and eurcamide have shown differential dynamic behavior, which confers the ligands shift from the binding pocket. Eurcamide initial rise in the trajectory level at 15–20 ns was about 3.1 ± 0.10 Å. Later, the plateau was depicted as steady until the end of the simulation around 50 ns. Similarly, behenic acid has shown a rise in the level of the plateau after 28 ns, which confirms the significant ligand, shifts out of the CDC2 pocket, and remained stable till the end of the MD run. Thus, the overall analysis of the MD simulation run suggests the ligand β-sitosterol has stable conformation into the binding pocket of CDC42. Comparatively, phenylacetic acid and palmitic acid have also been found to be stable. Euracamide and behenic acid ligands were observed to alter their position in their binding pocket of CDC42. The hydrogen bond formation plays a significant role during molecular interaction between the ligand and protein (CDC42). The term hydrogen bond donor and acceptor during hydrogen bonding indicate hydrogen atom from the donor and the acceptor with lone pair of electrons. From the MD simulation run, it was observed that β-sitosterol and palmitic acid shared a maximum of eight hydrogen atoms throughout the run. Phenylacetic acid has shared a maximum of four hydrogen bonds throughout the simulation run. Overall, the number of hydrogen bond donors, as well as acceptors, are within the range for β-sitosterol and palmitic acid (Figure 3B).

### STRING analysis

**FIGURE 4:**
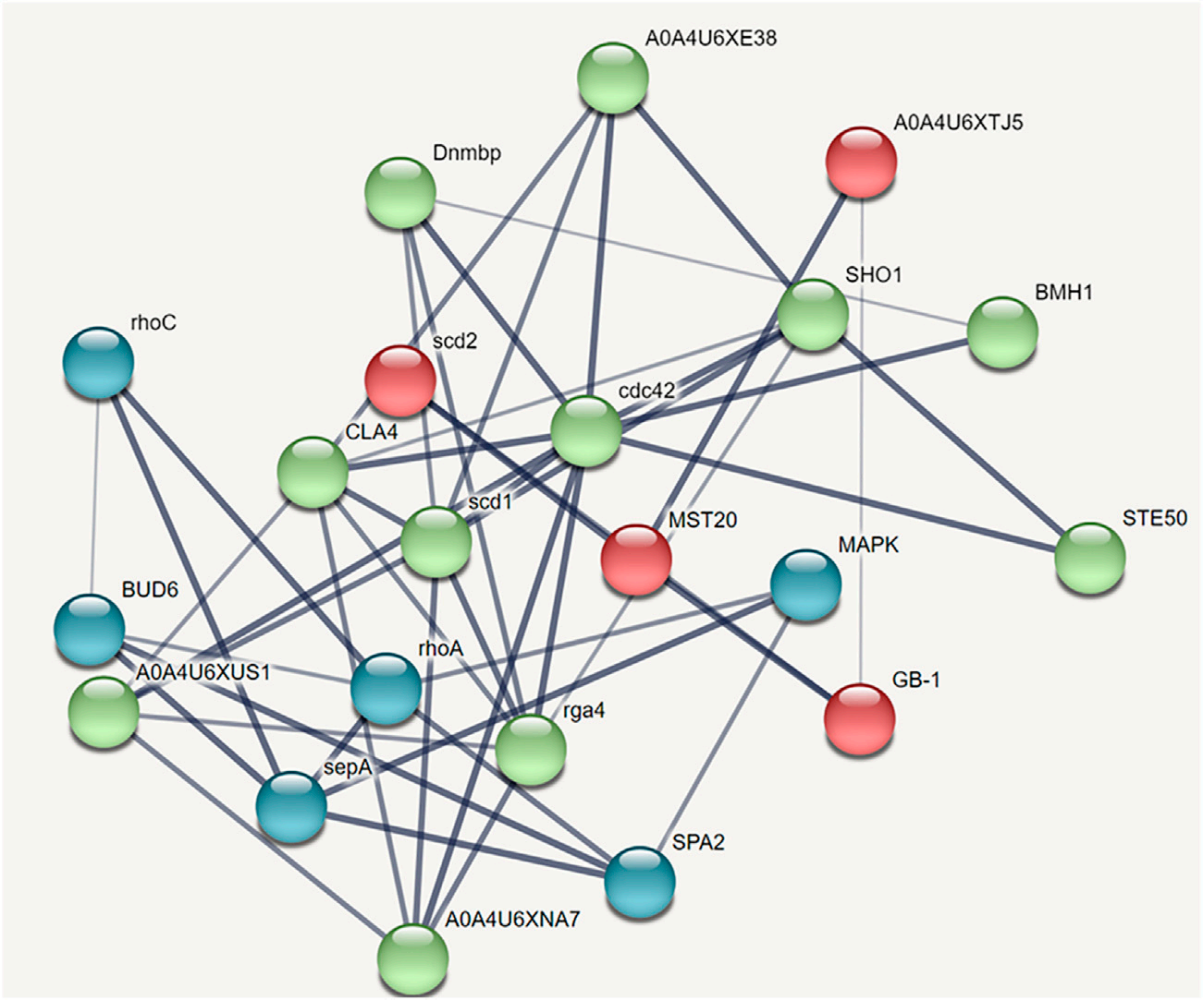
STRING network analysis displaying protein-protein interactions. Color nodes represents query proteins and first shell of interactions. Red color node represents cluster 1, green color represents cluster 2 and blue color represents cluster 3.

The protein-protein interaction network predicted through STRING analysis is shown in Figure 4. The network comprises 21 nodes; 133 edges; 12.7 average degree nodes; 0.812 average local clustering coefficient; 31 expected edges with protein-protein interaction enrichment values less than 1.0e^−16^. In Figure 4, color nodes represent the query protein and first shell of interactors while the white nodes represent the second shell of interactors. The empty nodes indicate proteins with an unknown 3D structure. The network edges represent the confidence mode in which the thickness of the line indicates the strength of the data support. From Figure 4, it is clear that there is no 3D structure available for the first and second shell interactors. Hence, further studies are required to understand the complex mechanism and detailed functions of the CDC42 homolog in establishing pathogenicity and diseases in plants.

### Clusters identification through MCODE analysis and GO classification

**FIGURE 5:**
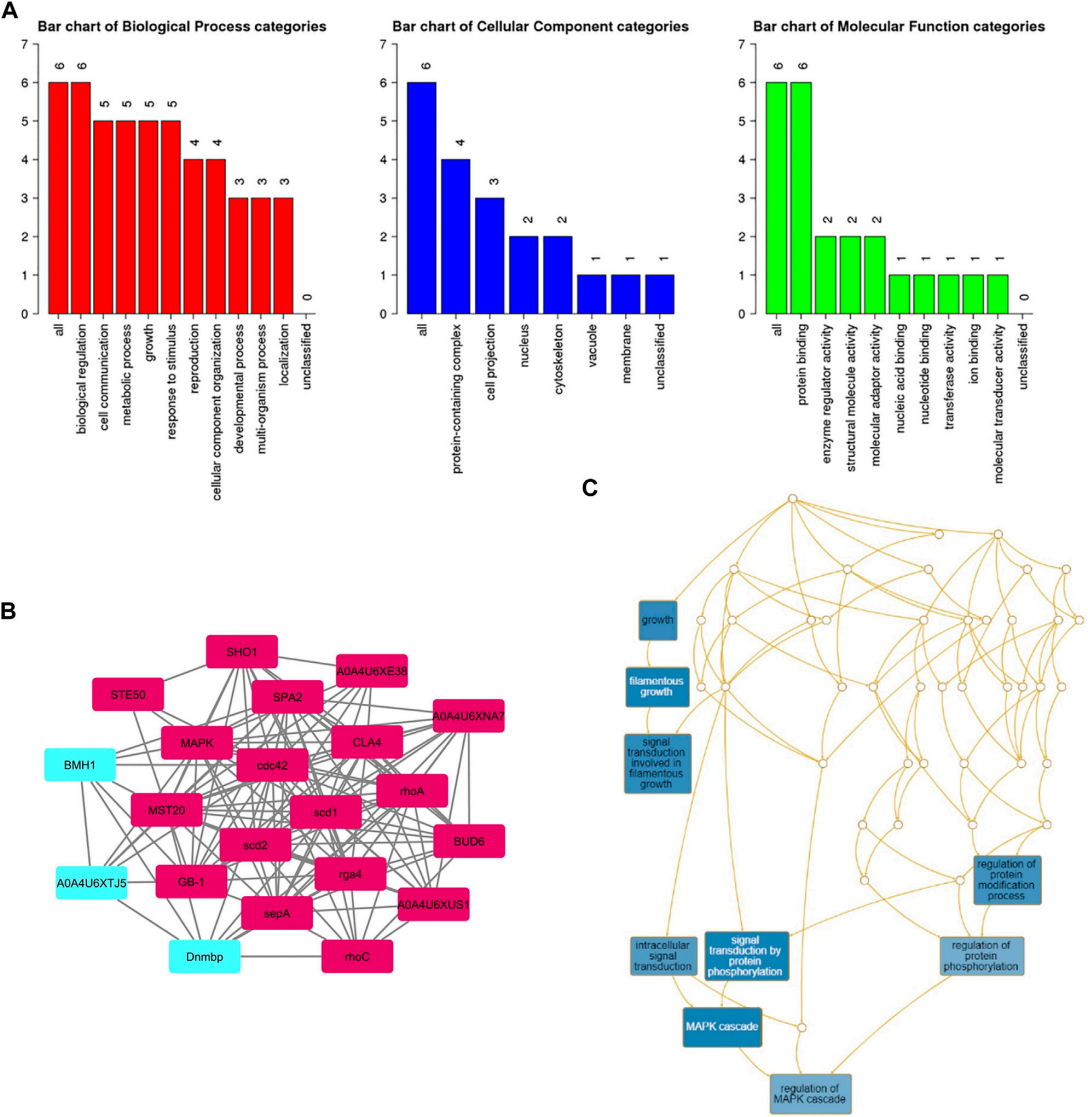
**(A)** Gene ontology analysisrepresents role of proteins in different process such as biological process, cellular and molecular function. The number of proteins involved in different process were represented above each bar. **(B)** node attribute enumerator analysis using MCODE available with Cytoscape (Maroon color subnetwork- 1; Cyan subnetwork-2); The subnetwork 1 is occupied with maximum numbers of proteins and subnetwork 2 is occupied with 3 proteins**(C)** Subnetworks - three clusters are highlighted in blue and the functional involvement of three clusters are represented in white font.

**FIGURE 6:**
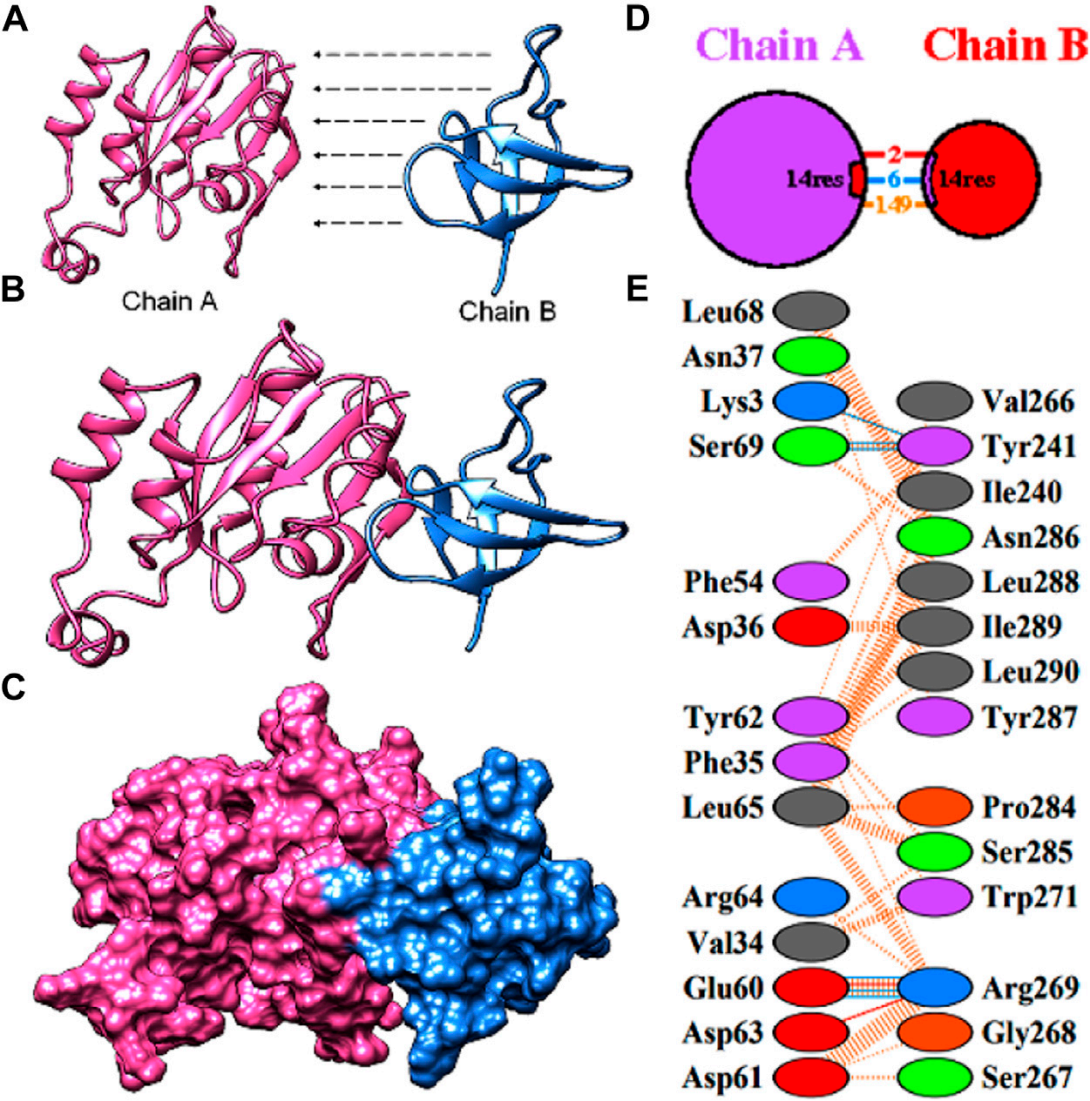
Protein-protein docking of the CDC42 with SHO1 from *C. gloeosporioides* **(A)** model proteins of CDC42 (pink) and SHO1 (blue). **(B)**and **(C)** interaction of the model proteins **(D)** pictorial representation of the interaction model and number of interactions **(E)** residues involved in interaction.

A subnetwork was constructed and the result was visualized through Cytoscape. CDC42 has shown interaction with SHO1, STE50, SPEA2, A0A4U6XE38, MAPK, CLA4, A0A4U6XNA7, MST20, SCD2, SCD1, RHOA, GB-1, SEPA, RHOC, A0A4U6XUS1, BUD6, RGA4, BMH1, A0A4U6XTJ5, and DNMBP. Further functional enrichment and Gene ontology analysis performed through the WEB-based GEneSeT AnaLysis Toolkit depicting three fundamental categories were presented in Figure 5A. The three main categories are biological process, cellular components, and molecular function. MCODE provides a real-time cluster assessment quality. The node attribute enumerator provides a numerical summary of node attribute values as shown in Figure 5B. Node attribute that is available for the loaded network is shown in box-1 which contains 15 nodes and 94 edges. The members of the clusters are represented in red. Exploration of clusters is shown in box-2 which contains 3 nodes and 3 edges. The members of the clusters are represented in red. The node scoring the highest value in the cluster is referred to as the seed. It is the node from which the cluster was derived, and it is represented in squares, and other cluster members are represented in circles. Edges indicating the interactions are represented in blue while the edge directionality is represented by arrows. New sub-clusters formed from the main cluster is shown in Figures 5C,D. The GO Slim summary is based on 6 unique Entrez gene IDs including BMH1, CLA4, SHO1, SPA2, STE50, and CDC42. Among 6 unique Entrez gene IDs, 6 IDs are annotated to the selected functional categories and also in the reference list, which are used for the enrichment analysis. All the genes mentioned above are predicted to play an important role in the MAP kinase pathway. The enrichment analysis revealed that the gene is mainly involved in filamentous growth, signal transduction (by protein phosphorylation), and MAPK cascade. Altogether, the KEGG enrichment analysis revealed the association of the MAPK signaling pathway. The enriched gene set for the MAPK signaling pathway was found to have a *p*-value of 1.1214^e−8^; an enrichment ratio of 45.46 The false discovery rate for the network was predicted as 0.00004 for a gene set size of 114 (Table 4). The protein sequence of SHO1 of *C. gloeosporioides* with the accession id A0A1B2LQ50 was obtained from Uniprot database. The sequence alignment of target and template sequence (yeast SHO1) with the sequence coverage of 61.02% was used for modelling the protein. Interaction analysis of CDC42 and SHO protein has shown 14 residues from each protein have good binding affinity. H-bond (6), non-bonded 149) and 2 salt bridges were established between the drug target (CDC42) and SHO1 (Figure 6).

**TABLE 4:** KEGG enrichment analysis for enriched gene set.

GeneSet	Description	Size	Overlap	Expect	Enrichment ratio	pValue	FDR	Gene symbol
sce04011	MAPK signaling pathway	114	5	0.109986	45.46053	1.12E-08	4.02E-05	BMH1; CLA4; SHO1; SPA2; STE50
GO:0030447	filamentous growth	135	5	0.130246	38.38889	2.64E-08	4.02E-05	BMH1; BUD6; SHO1; SPA2; STE50
GO:0000165	MAPK cascade	42	4	0.040521	98.71429	2.67E-08	4.02E-05	CLA4; SHO1; SPA2; STE50
GO:0023014	signal transduction by protein phosphorylation	47	4	0.045345	88.21277	4.25E-08	4.80E-05	CLA4; SHO1; SPA2; STE50
GO:0040007	growth	177	5	0.170767	29.27966	1.04E-07	9.36E-05	BMH1; BUD6; SHO1; SPA2; STE50
GO:0001402	signal transduction involved in filamentous growth	13	3	0.012542	239.1923	1.42E-07	1.07E-04	BMH1; SHO1; STE50
GO:0031399	regulation of protein modification process	212	5	0.204534	24.44575	2.56E-07	1.66E-04	BMH1; CLA4; SHO1; SPA2; STE50
GO:0035556	intracellular signal transduction	252	5	0.243126	20.56548	6.10E-07	3.45E-04	BMH1; CLA4; SHO1; SPA2; STE50
GO:0043408	regulation of MAPK cascade	32	3	0.030873	97.17188	2.45E-06	0.00123	CLA4; SHO1; SPA2
GO:0001932	regulation of protein phosphorylation	133	4	0.128316	31.17293	2.90E-06	0.001312	CLA4; SHO1; SPA2; STE50

## Discussion

Anthracnose disease occurrence in cassava can lead to total economic loss for the cultivators by damaging the total harvest into the rotting waste. Anthracnose disease occurs in plants due to fungal species of the genus Colletotrichum. The species such as *C. fructicola*, *C. gloeosporioides*, *C. tropicale*, *C. theobromicola*, *C. siamense*, *C. brevisporum,* and *C. plurivorum* are the most common group of plant pathogens that are responsible for diseases on many plant species. Infected plants with fungal strains develop dark patches and lesions on stems, leaves, or any parts of the plant. The lesions occurring on the infected region (leaves, stem) appear to be the gelatinous masses of spores. The fungi during infection come in close contact with the adherence of the spores. The germination starts after several hours with favorable conditions such as temperature. During the suitable temperature, the fungi germinate the conidia and produce the germ tubes. Fungi develop appressorium and arrest the elongation of the germ tube. The penetration of appressorium promotes turgor pressure and fungi colonize the plant tissues, which appear like a canker.

The role of genes in the penetration and development of infection has not been revealed so far in cassava. However, mitogen activator (PMK1, MPS1), ATPase (PDE1), Tetraspanins (PLS1), and fungal effector genes were reported as important genes for infection in rice blast fungus. CDC42, an important protein essential for cell division and cell cycle from cassava, was investigated as the molecular target for the present study. From the sequence analysis, CDC42 was revealed with 190 amino acids and belongs to the small GTPase family. In fungi, the presence of the small GTPase is essential for both beneficial and pathogenic interaction with the plant system. The small GTPases of cassava are characterized as Rho family, essential for the formation of virulence, a fusion of pathogen with plant cell, and production of reactive oxygen species (ROS). Thus, the protein with three motifs is structurally essential for the GTPase activity. In absence of the three-dimensional structure, the protein was modeled based on sequence similarity. The CDC42 protein from *C. gloeosporioides* has shown 70.83% homology with CDC42 of humans with query coverage of 98%. The modeled structure was validated and used for further docking studies. Generally, *Bacillus* sp. is considered a promising source for bioactive secondary metabolites. Therefore, in the present study molecular docking was carried out with the bioactive compounds (erucamide, behenic acid, palmitic acid, phenylacetic acid, and β-sitosterol) of *B. megaterium.* There are several pieces of evidence for the compounds identified from *B. megaterium* as lead molecules. Erucamide identified from radish leaf has shown preventive effects against memory deficits related to Alzheimer’s disease by modulation of cholinergic function. *In vivo* experiments have shown that erucamide has biological activity such as stimulation of angiogenesis, augmentation of neovascularization in regenerating skeletal muscle, and anti-depressive effects (Kim et al., 2018). Similarly, behenic acid-based nano micelles were prepared with dextran as the combination to deliver itraconazole as a drug. The nano micelles were used as anti-leishmaniasis for targeting the parasite (Shahriyar et al., 2021). The saturated fatty acid (C:16), the palmitic acid, selected as the lead molecule in the present study has been deeply investigated previously (Lee et al., 2009) as an antiviral agent. Palmitic acid specifically binds to the CD4 and prevents the entry of the HIV-1 virus. Moreover, beta-sitosterol has been reported from plants and is known for anticancer effects against several cancers such as breast and ovary, prostate, lung, stomach, and liver. In addition, the compound can significantly inhibit several pathways, cell signaling, apoptosis, angiogenesis, and inflammation (Bin Sayeed and Ameen, 2015). Phenylacetic acid and its derivatives were extensively used in the preparation of drugs that can be used for several ailments. Diclofenac is used as a medication for the treatment of pain and inflammation (Gan 2010). Apart, the previous report indicated that the purified components possess significant antimicrobial activity against plant pathogens such as *A. tumefaciens* (T-37), *Erwinia carotovora* (EC-1), and *Ralstonia solanacearum (*RS-2) (Xie et al., 2021). Among the five components investigated against plant pathogens, β-sitosterol, behenic acid, and phenylacetic acid displayed significant antimicrobial activity. Β-sitosterol showed a very low minimum inhibitory concentration (15.6 μg/ml) against *R. solanacearum* RS-2 (Xie et al., 2021). Thus, the five compounds of choice used for the present study have already been investigated for several alignments. Mostly, these compounds were reported from different plant species; however, in the present study, the compounds identified from *B. megaterium* were used for investigation. The compounds were docked into the binding pocket of the CDC42 of *C. gloeosporioides.* β-sitosterol and phenylacetic acid showed the highest dock score of −10 kJ mol ^−1^. The next top hit obtained was palmitic acid (−9.4 kJ mol ^−1^) followed by behenic acid (−9.2 kJ mol ^−1^) and erucamide (−9.2 kJ mol ^−1^). From the binding energy analysis, it was observed that β-sitosterol obtained the highest binding affinity of −41.51(Kcal/mol).

To study the stability during their interaction, molecular simulation was performed and the results showed that the compounds were stable throughout the simulation. For a comprehensive analysis of the docked protein-ligand complex, a molecular dynamics simulation was carried out. It is the most powerful technique to study the conformational changes taking place at the atom level. Therefore, molecular dynamics simulation was performed for some time of 50 ns for all atom-docked protein-ligand complexes. The result showed b-sitosterol with stable conformation compared to the other docked complexes. The results are evidence that the docked protein- β-sitosterol complexes are highly stable for the entire period of 20–50 ns. Furthermore, RMSD plot analysis showed slight modification in the position, indicating the stable association and interactions between the ligand and the protein molecule. Also, the ligand has maintained a maximum of eight hydrogen bond interactions throughout the MD run. Thus, the stability of the ligand with CDC42 shows the ligands can be extended further as a biological agent to treat pathogenesis against *C. gloeosporioides*. Additionally, studies have shown that β-sitosterol has already been used for the treatment of various diseases due to its potent properties such as antinociceptive, anxiolytic and sedative effect, anticancer, antimicrobial, immunomodulatory, hepatoprotective, and wound healing effects. The chemical has already been approved by FDA and is a safe nutraceutical with no deleterious effects (Babu and Jayaraman, 2020).

**FIGURE 7:**
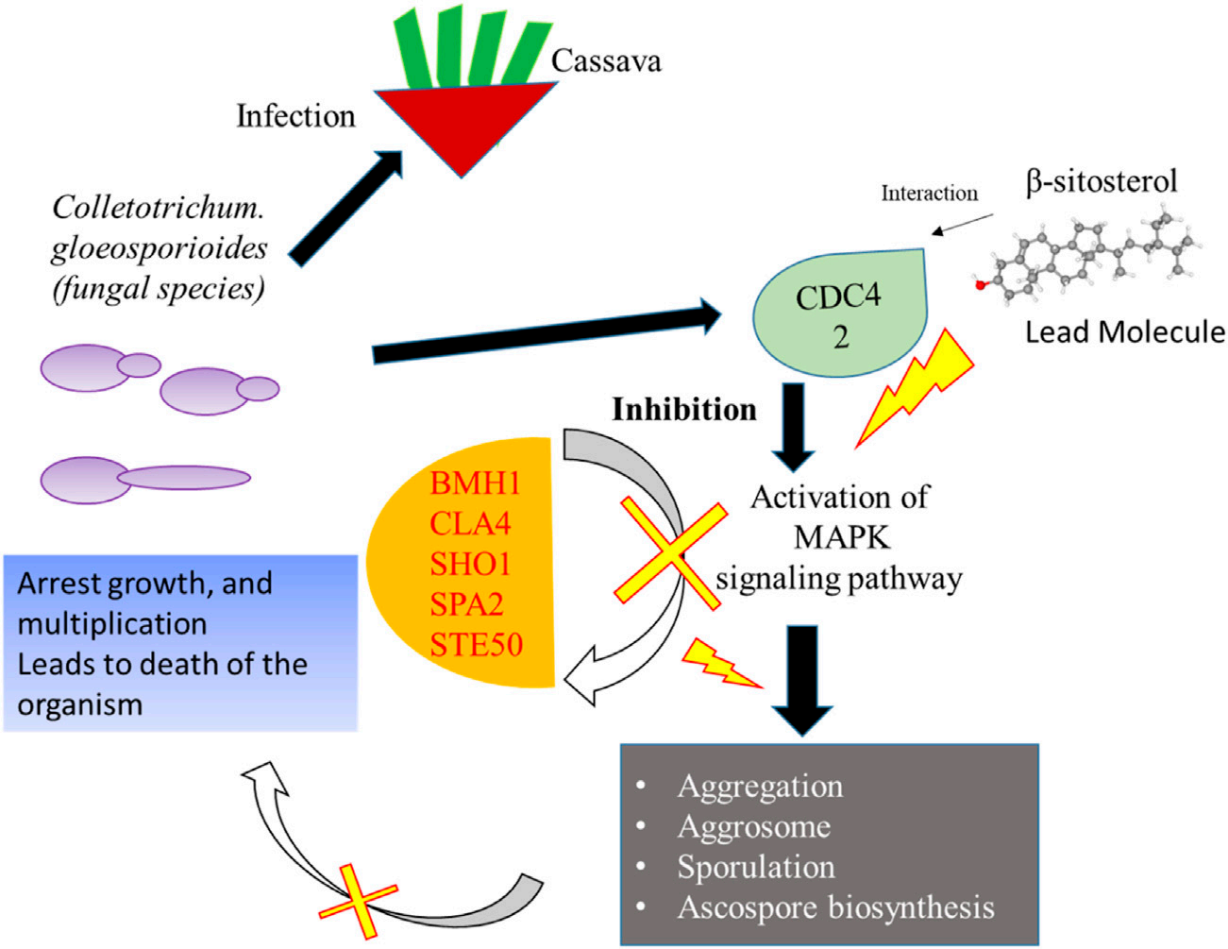
Details of the overall mechanism representing CDC42 from *C. gloeosporioides* involved infection and inhibition of the signaling pathway using lead molecules.

Network-based approaches provide a deep insight to understand the biological process during the pathogenesis of *C. gloeosporioides*. The interacting partner revealed through the PPI network will pave the way to investigate the cellular activity, protein localization, and complex biological function of the protein. Besides, 20 genes have shown interaction with CDC42 and from the MCODE statistical analysis, two clusters were identified one with 15 nodes and 94 edges and the second cluster with 3 nodes and 3 edges. Furthermore, the functional enrichment analysis has revealed the BMH1, CLA4, SHO1, SPA2, and STE50 as the important genes involved in the MAPK signaling pathway. The protein BMH1 has shown to play important role in aggregation, and arrangement to form aggresomes. Additionally, BMH1 is involved in spore formation, sporulation, and ascospore biosynthesis. The CLA4 is very essential for imparting *Cladosporium* resistance in the organism. SHO1 protein, the osmosensor present in the plasma membrane of *C. gloeosporioides* activates the high osmolality glycerol (HOG) of the MAPK signaling pathway in response to high osmolality. SHO1 protein is found in bud and bud neck region of the fungal pathogen. Protein docking interaction reveals SHO1 and CDC42 has established binding affinity. Hence it is envisaged that inhibition of CDC42 can significantly prevent the signalling and inhibit the growth and development of the fungal pathogen. SPA2 perhaps a cytoskeletal protein is involved in pheromone-induced morphogenesis, budding, invasive filamentation growth, regulation of hyphal growth, cellular shape, and reproduction of *C. gloeosporioides*. STE50 protein has shown to play a significant role in signal transduction during filamentous growth, osmosensory signaling MAPK cascade thereby arrest the growth during conjugation. Thus, the identified interacting partners of CDC42 are involved in the MAPK signaling pathway essential for growth, and virulence regulation in *C. gloeosporioides*. Therefore, from the present study, it is revealed that targeting CDC42 can impart the interaction network, prevent filament production, and arrest the reproduction in *C. gloeosporioides* (Figure 7).

## Conclusion

Infections caused by *C. gloeosporioides* in cassava are very serious to impair, leading to economic damage to the cultivators. To date, there are no clear details about the type of infection and mode of transmission, and pathogenesis of the fungal pathogen. Hence, in the present study, CDC42 protein involved in cell division and cell cycle of the pathogen was selected as the target. Modeling of the protein revealed the key residues playing the functional role of the protein. The protein was targeted with five active compounds from *B. megaterium*. Interaction of β-sitosterol and phenylacetic acid with the key residue of CDC42 revealed that ligands could have a potential role in the inhibition of functional proteins that are involved in growth. Further PPI network constructed for CDC42 revealed that targeting the protein could impart MAPK signaling pathway. Additionally, targeting the interacting partner could also prevent the growth, filamentation, and hyphal growth which is essential for virulence regulation. However, further experimental insight can pave a way for preventing *C. gloeosporioides* mediated infection in cassava.

## Data Availability

The original contributions presented in the study are included in the article/Supplementary Material, further inquiries can be directed to the corresponding author.

## Supplementary material

Supplementary Figure 1 Structural validation through Saves and ProSA program (A) Verify3D (B) Ramachandran plot for model obtained from swiss model server and modeller (C) ProSA score for the template, 2NGR (D) ProSA score for target, CDC42.

Supplementary Figure 2 superimposition of model from Swissmodel (blue) and modeler (pink).
