# Supplementary material for: Investigation of bioactive compounds from Bacillus sp. against protein homologs CDC42 of Colletotrichum gloeosporioides causing anthracnose disease in cassava by using molecular docking and dynamics studies
Source: Front Mol Biosci. 2022 Sep 23;9:1010603. doi: 10.3389/fmolb.2022.1010603 (PMC9537347; doi:10.3389/fmolb.2022.1010603)
Supplement: Supplementary file 1 [file DataSheet1.docx]

**Investigation of bioactive compounds from Bacillus sp. against protein homologs CDC42 of *Colletotrichum gloeosporioides* causing anthracnose disease in cassava by using molecular docking and dynamics studies**

**Supplementary Figure 1:** Structural validation through Saves and ProSA program (A) Verify3D (B) Ramachandran plot for model obtained from swiss model server and modeller (C) ProSA score for the template, 2NGR (D) ProSA score for target, CDC42

(A)

**
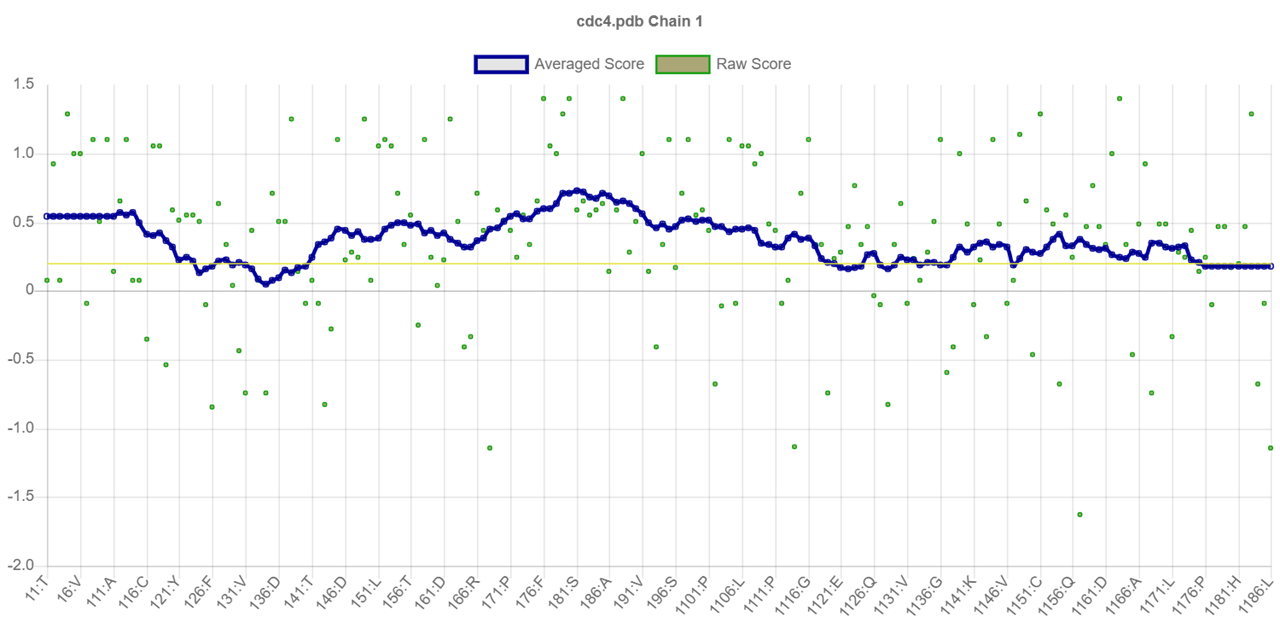
**

**(B)**

**
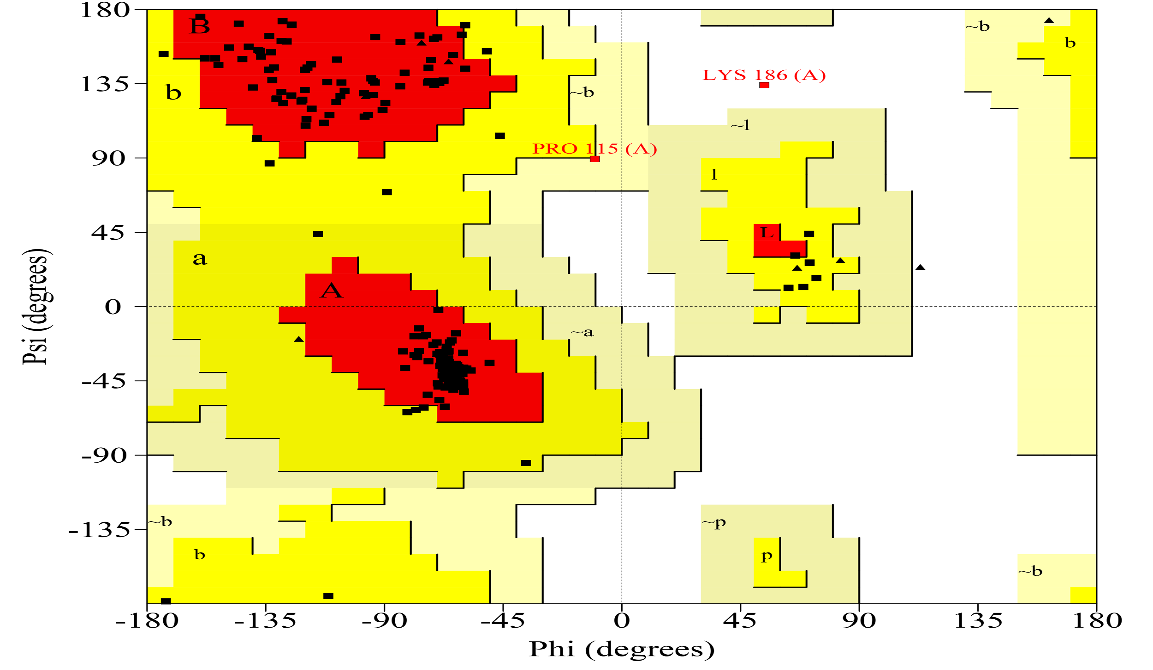
**

**
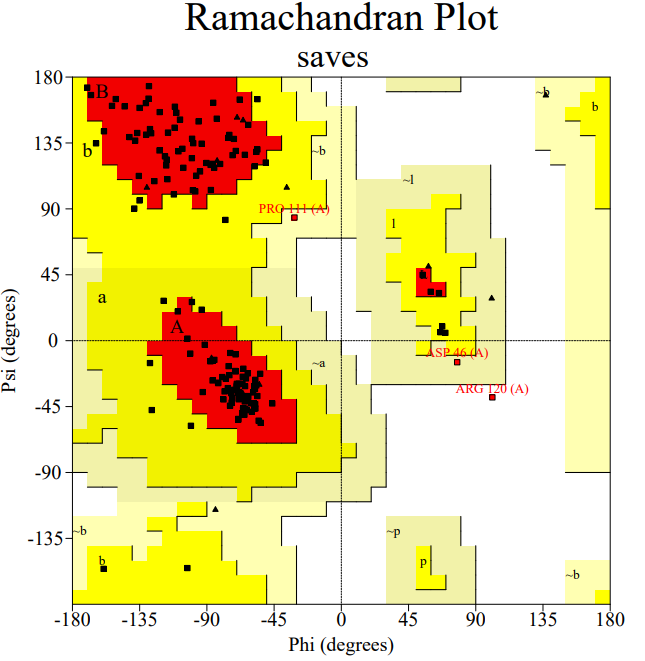
**

**
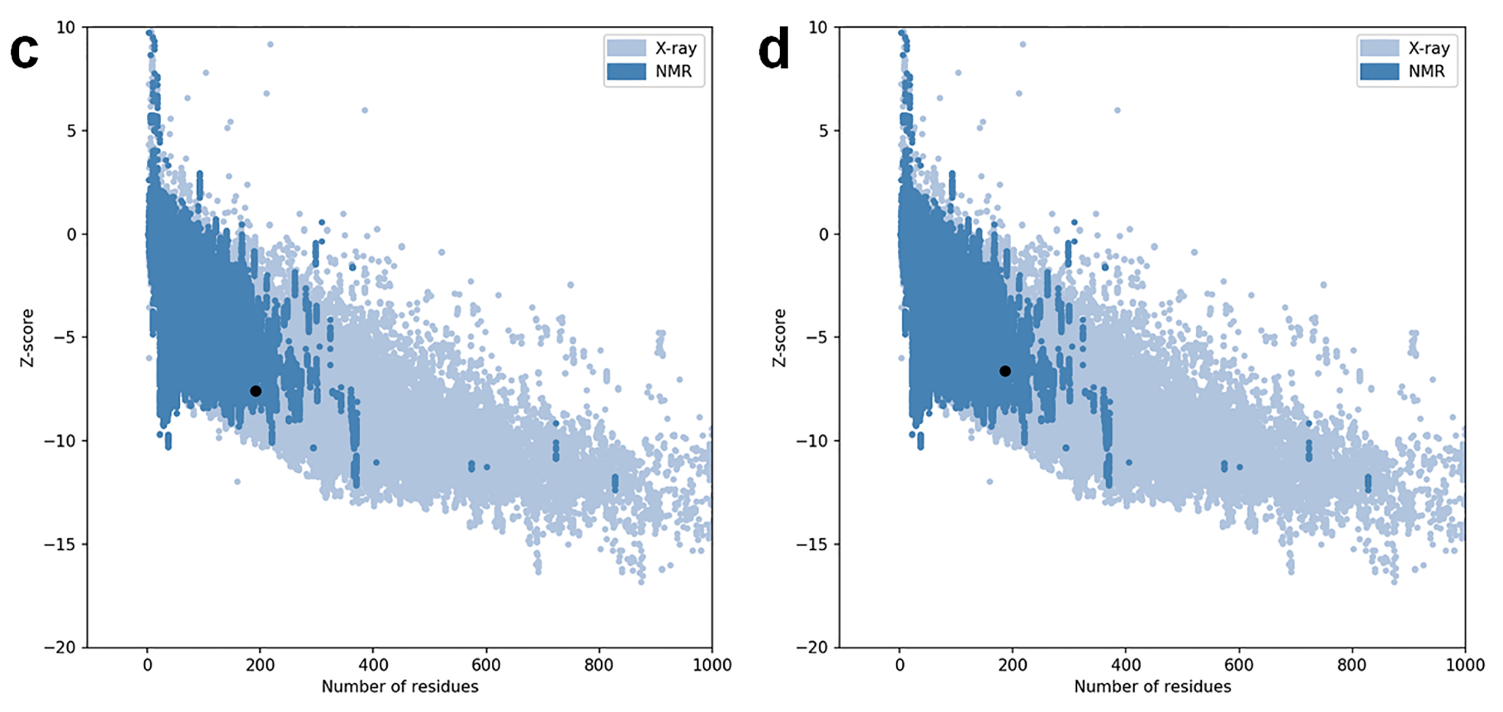
**

**Supplementary Figure 2 : superimposition of model from Swissmodel (blue) and modeler (pink)**

**
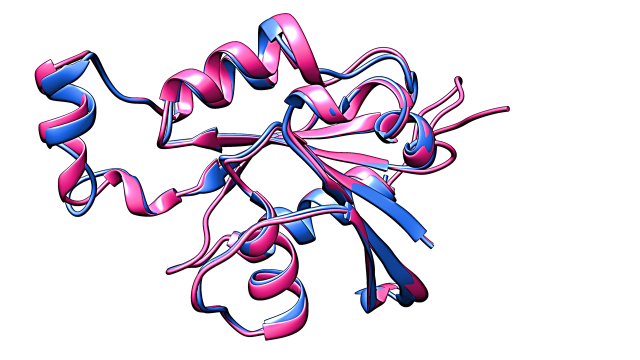
**
